# Stopping Smokeless Tobacco Use: A Call to Action

**DOI:** 10.3389/fpubh.2021.601890

**Published:** 2021-05-28

**Authors:** Donald Reed, Elaine Bowen, Becca Fint-Clark, Brent Clark, Nila Cobb, Kathy M. Danberry, Zona Hutson, Stephanie Lusk, Jason Rine, Natasha Robinson

**Affiliations:** ^1^McDowell County Commission on Aging, Liberty University, Lynchburg, VA, United States; ^2^Extension Service, West Virginia University, Morgantown, WV, United States; ^3^West Virginia (WV) Division of Tobacco Prevention, Charleston, WV, United States; ^4^Athletic Academic Support, Central State University, Wilberforce, OH, United States

**Keywords:** youth, smokeless tobacco, 4-H, oral health, extension service

## Abstract

In the United States, single smokeless tobacco use continues to increase in conjunction with the dual use of smokeless tobacco and other nicotine products. Problematically, much of the tobacco prevention literature and funding inundates tobacco users with smoking tobacco information while neglecting to provide them any information about smokeless tobacco. Meanwhile, American tobacco companies continually market new and dissolvable tobacco products targeted at non-smokers. New data suggests that smokeless tobacco use is, also, increasing in West Virginia and, in order to address this increased use, the West Virginia Extension Service recently partnered with the Division of Tobacco Prevention in the West Virginia Department of Health and Human Resources to develop a comprehensive spit tobacco curriculum for West Virginia students between third and sixth grade. This article details the development and assessment of the spit tobacco prevention curriculum and the resulting report from the initial pilot of the program. The curriculum was piloted across six counties with the participation of schools, after-school programs and 4-H clubs. After implementation, survey results demonstrate that youth have increased awareness of the health effects of smokeless tobacco. Throughout the article, we explore West Virginia's Cooperative Extension Service's response to this emerging public health issue and release a call to action for the National Cooperative Extension Services to join us in spit tobacco prevention.

## Introduction

This article illustrates the development, piloting, evaluation, and implementation of the Stop Spit Tobacco Curriculum in West Virginia. We highlight both the dangers of spit tobacco and the potential effects of spit tobacco on communities. The West Virginia Extension Service partnered with the Division of Tobacco Prevention in the West Virginia Department of Health and Human Resources to develop a comprehensive spit tobacco curriculum for West Virginia students in Grades 3–6. We conclude by putting out a call to action to all American Extension Professionals to prevent spit tobacco use in their own communities.

The term *spit tobacco* refers to tobacco that is not burned, is used orally, and produces the need to spit as by-product—i.e., chewing tobacco and snuff. The term *smokeless tobacco* refers to smokeless and spitless tobacco products—i.e., snus and dissolvable tobacco products. The term *spit and smokeless tobacco* refer to all tobacco products that are not ignited with fire to use.

## The Dangers of Spit Tobacco

According to the National Summit on Smokeless and Spit Tobacco, “Smokeless and spit tobacco (SST) use is a rapidly-evolving public health threat requiring greater leadership, a shift in research and funding priorities, and local public action” (2015). While there have been many public interventions in smoking tobacco access and use—namely: Food and Drug Administration (FDA) regulations, excise taxes, public service announcement campaigns, and cessation-based phoneline services—there are, currently, no strategies to help communities address Smokeless Spit Tobacco (SST). With the development of dissolvable tobacco products that attract both curious young people and longer-term smokers, we need programs centered around SST research and prevention more than ever before. Research conducted by the National Summit on Smokeless and Spit Tobacco has demonstrated that SST products can not only cause oral cancer, gum, and tooth disease, but can also contribute to other forms of cancer, heart disease, and strokes (1).

The harmful properties of spit tobacco have been well-documented, but many people continue to believe that SLT use is safer than cigarette use. SLT users, however, “run the same risks of gum disease, heart disease, and addiction as cigarette users, but [face] an even greater risk of oral cancer” (2). A 2015 analysis of the worldwide healthcare impact of SLT use estimates that SLT consumption by adults prior to 2010 resulted in more than 62,000 deaths from oral, pharyngeal, and esophageal cancer as well as the loss of an estimated 1.7 million disability-adjusted life years (3). In 2011, SLT sales in the United State totaled ~124.6 million lbs, a 2 million-lb increase from the 122.6 million lbs sold in 2010 (4). Additionally, tobacco use costs the United States billions of dollars in medical expenses and lost productivity (5).

SLT contains at least 30 carcinogen chemicals that are known to cause cancer, with tobacco-specific nitrosamines found to be the most harmful (6). This chemical forms during the growing, curing, fermenting, and aging of tobacco (6). While cancers caused by SLT are most likely to develop at the site where tobacco is held in the mouth (2), other places such as the tongue, cheek, gum, esophagus, and pancreas can also be affected (7). According to the American Academy of Otolaryngology (2), additional ingredients in SLT include polonium 210 (nuclear waste), n-nitrosamines (carcinogens), formaldehyde (embalming fluid), nicotine (an addictive drug), cadmium (a chemical in car batteries and nuclear reactor shields), cyanide (a poisonous compound), arsenic (a poisonous metallic element), benzene (a product used in insecticides and motor fuels), and lead (a chemical that causes nerve poisoning).

In addition to increasing an individual's risk of cancer, SLT has many harmful effects on teeth, gums, and the mouth. The sugar in spit tobacco, for example, can cause tooth decay (7), and the coarse particles in the tobacco can irritate the gums and scratch away tooth enamel. The product can cause periodontal disease that destroys soft tissue and bone support and which can lead to tooth loss. White patches and red sores, known as leukoplakia, may also appear in the mouth and have the potential to turn into cancer (7).

SLT has additional negative health effects on several body systems. Using the product during pregnancy increases the risk of early delivery and even stillbirth, and can affect the brain development of the fetus (8). SLT may even cause nicotine poisoning if children ingest it (9). Using SLT increases an individual's risk of death from heart disease or stroke because use of the product has been linked to increased heart rate and blood pressure (10).

## Health Disparities and West Virginian Statistics

“Health disparities are preventable differences in the burden of disease, injury, violence, or opportunities to achieve optimal health that are experienced by socially disadvantaged populations” (11). Such “populations can be defined by factors such as race or ethnicity, gender, education or income, disability, geographic location (e.g., rural or urban), and sexual orientation” (11). Such “health disparities are inequitable and are directly related to the historically and presently unequal distribution of social, political, economic, and environmental resources” (11).

“Health disparities result from multiple factors,” (also known as the social determinants of health) “including poverty, environmental threats, inadequate access to healthcare, inadequate healthcare, individual behavior choices, and education inequalities” (11). Citizens with less education are more likely to experience health risks that may include obesity and substance abuse. In reverse, research has correlated good health with academic success (11).

With exceptions for deaths due to influenza, pneumonia, and stroke, West Virginia has some of the highest rates of death due to preventative measures (12). The state also ranks among the bottom tier of states across the presented health risk factors, with even higher rates of obesity and high blood pressure within the state's black population (12). Across all populations within the state, the obesity rate exceeds 25%, nearly the worst in the nation.

According to the 2013 West Virginia Youth Tobacco Survey, West Virginia had the second-highest ranking for male smokeless tobacco use among high school students in the nation. The percentage of male smokeless tobacco users in West Virginia (15%) was significantly higher than the national percentage (6.7%). In 2011, 25.5% of youth Surveyed in West Virginia high schools reported that they were current users of smokeless tobacco, compared to 12.8% in the United States (13).

While the state is among those in the nation with the lowest rates of health insurance coverage and dental visits, it falls within the middle range of states for the other presented measures of preventive care. As comparable with other health disparities, tobacco-related disparities are, to some extent, caused and perpetuated by social determinants of health: scarcity of resources for the poor, environmental threats, and insufficient admission to healthcare, inadequate healthcare, individual conduct choices, cultural customs, and teaching inequalities.

## Linking to Tobacco Use Prevention and the 4-H Healthy Living Mandate

4-H is the United States of America's federal youth development program, which is administered by the land-grant university and college system. Since 1914, health and healthy living have been priorities of the 4-H youth development program of the United States Department of Agriculture and the Cooperative Extension System (22). In 1994, the national 4-H headquarters established the 4-H mission mandates of science, citizenship, and healthy living to provide a more cohesive system-wide approach to youth development programming across the country (22). The healthy living mission mandate focuses on the areas of health, healthy eating, physical activity, social/emotional health, alcohol awareness, tobacco awareness, drug use prevention, and injury prevention (22).

According to the National 4-H Council of Healthy-Living Logic Model for Prevention of Alcohol, Tobacco, and Other Drugs (ATOD), activities and curricula should target youth audiences (with a special focus on new and underserved audiences), families, staff, volunteers, community leaders, partner organizations, and collaborators. Educational efforts should provide tobacco cessation information, resources, and support to young people and their families; multi-component programs targeted to different developmental stages relating to ATOD intervention; opportunities to model non-use among young people with family and friends; community mobilization campaigns to prevent and reduce ATOD use; programs with multiple components such as using environmental changes, policy changes, social marketing campaigns, and curricula that meet ATOD prevention standards for skill-building and self-efficacy; and opportunities to involve families in meaningful ways (14).

According to the Campaign for Tobacco-Free Kids (CTFK)—a national advocacy organization working on public policy and education on the dangers of tobacco use—the vast majority of all initial tobacco use begins in high school. The CTFK recommends the following steps to create an environment that encourages tobacco prevention among young people: tobacco-free school policies; comprehensive tobacco prevention education; involvement of parents and families in school efforts to prevent tobacco use; help for tobacco-using staff and students to quit; interactive tobacco-free projects for students; and the adoption of a school policy that prohibits the acceptance of funding, curricula, or other materials from any tobacco companies (15). We used the recommendations of the CTFK when developing the Stop Spit Tobacco Curriculum. The CDC recommends that prevention educators “provide instruction about the short- and long-term negative physiologic and social consequences of tobacco use, social influences on tobacco use, peer norms regarding tobacco use, and refusal skills” (16). Successful programs to prevent tobacco use address multiple psychosocial factors related to tobacco use among children and adolescents. By educating our youth about spit tobacco and promoting prevention, the curriculum specifically follows the 4-H mission mandate.

According to the CDC, there are many factors that influence young people to begin using tobacco, which include, exposure to tobacco advertising, low self-image, lack of support or involvement from parents, the normalization of tobacco use within peer groups, and a lack of self-efficacy that contributes to resisting influences of tobacco use (17, 18). The CDC also reported that in 2016, 7.2% of middle school students reported using any form of tobacco, and 2.2–4% of high school male students reported using smokeless tobacco on or at least 1 day during the past 30 days. Our Stop Spit Tobacco prevention curriculum was geared toward students in third-sixth grade and our objective was to instill them with tobacco resistance skills for middle school, in line with the CDC's recommendations. 4-H does have a smoking prevention curriculum, Health Rocks, however, its main topic area is smoking. The statistics above show that a more targeted curriculum geared toward spit tobacco is needed (19).

Our curriculum utilized six different lessons to address spit tobacco prevention giving participants the education, media literacy, confidence to say no, and ability to get civically involved in spit tobacco policies. These lessons meet the mission mandates of 4-H. The innovation of linking the 4-H network with tobacco prevention effort has the potential to create a nationwide movement around the issue.

## Project to Stop Spit Tobacco

Over the past 3 years, a team of Extension Agents and a Curriculum Specialist have worked with West Virginia University Communications, the West Virginia University Prevention Research Center, and the West Virginia Division of Tobacco Prevention to complete a full Stop Spit Tobacco Curriculum. The curriculum includes speaker scripts, background information, handouts, resources, and evaluation tools. In 2014, the team worked individually to author six lessons: (1) The Body: Tobacco Bad Effects, (2) Addiction: Hard Cycle to Break, (3) Cost: Can You Afford to Spit? (4) Media: Promotion of Spit Tobacco – A Deadly Influence, (5) Say No: To Spit Tobacco, and (6) Advocacy: Your Voice Counts. Other pieces of the curriculum included a preface, an introduction, and a letter that was sent home to parents giving them tips on how to talk to their children about spit tobacco. The curriculum includes multiple hands-on activities in each section that demonstrate the topics of each lesson in a way that young people in Grades 3–6 would understand. Before the curriculum was distributed for wider use, it was piloted by Extension Agents in their home counties. Feedback was provided by the students who took part in the pilot lessons and the lessons were edited based on the student feedback.

Depending on how many activities the teacher chose to use, each lesson took ~30–45 min to complete. Lessons were delivered over a 6-week period to students or 4-Her's. A typical lesson plan included a list of the required materials, learning objectives, topic background, introduction, learning activities, lesson conclusion, preparation notes, and a list of which national content standard the lesson met. National content standards were introduced to persuade teachers and school administrators to use the curriculum in their classrooms. All lessons came with a script to follow to encourage active discussion throughout the lesson. An appendix was included to ensure that all material was readily available to teachers, club leaders, or other facilitators to use. This is the final draft of the curriculum after feedback was provided by the pilot runs of the Stop Spit Tobacco Curriculum.

Lesson one, “The Body: Tobacco Bad Effects,” was designed to educate students about the dangers of spit tobacco by showing them the ingredients within the product and explaining how the ingredients negatively impact the body, both in the short and long term. The second lesson, “Addiction: Hard Cycle to Break,” encouraged students to take part in activities about the perils of spit tobacco addiction. The activities illustrated how difficult it is to break away from addiction, taught ways that make it easy to say “no” to spit tobacco, and provided better alternatives to using spit tobacco. Lesson three, “Cost: Can You Afford to Spit?,” was created to help young people assess the costs related to spit tobacco use and to learn about the value of spending money wisely by determining the intrinsic value of items and activities compared with the value of spit tobacco. The next lesson, “Media: Promotion of Spit Tobacco – A Deadly Influence,” helped students analyze how messages from the media and other sources influenced their health behaviors. Lesson five, “Say No: To Spit Tobacco,” was designed to help young people manage peer pressure and make thoughtful health decisions by using verbal and non-verbal skills to say “no” to friends. The last lesson, “Advocacy: Your Voice Counts,” taught students about advocacy—how it works and why it is important—through hands-on learning experiences and a stop spit tobacco advocacy project. By focusing on these topics, we are providing students with the skills that they need to make healthy decisions regarding spit tobacco while, also, encouraging them to become involved citizens in tobacco prevention. Letters that were sent home to parents opened the door for them to be involved in an ongoing conversation with their children about spit tobacco, and, in the meantime, students created media showing the consequences of using spit tobacco and were encouraged to become advocates in spit tobacco prevention. Ultimately, the educational material in the curriculum reiterated the physical and financial costs of spit tobacco. In Table 1 below, we detail each lesson name, lesson objectives, and a sample activity.

**Table 1:** Curriculum Lessons.

**Stop spit tobacco lesson overviews**		
**Name of lesson**	**Lesson objectives**	**Sample activity**
Lesson One, The Body: Tobacco Bad Effects	• Students gain an understanding of the many toxic materials found in spit tobacco. • Students understand the short-term negative impacts of spit tobacco use on the body and be introduced to some of the long-term effects.	What's in that Can? – Activity showing ingredients in spit tobacco
Lesson Two, Addiction: Hard Cycle to Break	• Students understand that addiction to tobacco makes it very hard to stop the use of spit tobacco products and the ways addiction itself is harmful. • Students understand some of the external pressures that lead to experimenting with spit tobacco and some of the outside supports that help students say no to experimentation with tobacco. • Students discover positive alternate activities they could practice instead trying tobacco.	Tied Up–Students have one string wrapped around wrist and break it. Each time they use spit tobacco another string is added, to show strength of addiction with each use.
Lesson Three, Cost	• Students understand what it costs to use spit tobacco for 1 year. • Students learn how the “value” of using spit tobacco compares to using other items and engaging in other activities.	The Cost of Spit Tobacco– Students count total amount of money in envelope equaling cost per year of spit tobacco use.
Lesson Four, Media: Promotion of Spit Tobacco – A Deadly Influence	• Students recognize the influence of media upon their purchasing decisions. • Students recognize marketing strategies used in smokeless tobacco advertising. • Students learn media evaluation skills to help them better determine the validity of messages used in advertising smokeless tobacco products.	Creating Truthful Spit Tobacco–Students make an ad showing real impact of spit tobacco use.
Lesson Five Say No: To Spit Tobacco	• Students learn about the conditions that help someone resist using spit tobacco or participating in other risky/unhealthy behaviors. • Students understand that one cannot get away from pressure from friends or others trying to persuade us to do many things, but one can have strategies that will help to combat pressure from friends to participate in unhealthy behaviors. • Students learn certain facts about verbal and nonverbal refusal skills so they will be able to use the skills with confidence when they are being pressured to try risky behaviors by their friends.	Discovering Positive Body Language – Students learn how body language can impact the way their message is received.
Lesson Six Advocacy: Your Voice Counts	• Students learn about the functions of advocacy and recognize the power of young people as effective advocates. • Student learn ways to determine a cause and how to identify who the most effective people would be to receive the advocacy message. • Students work individually or in groups to develop messages about the dangers of spit tobacco to children.	The Power of Postcards- Students write postcards to community leaders advocating for policy change on spit tobacco (i.e., use in public).

Once the initial project was accomplished, the team of extension agents completed a pilot of the curriculum with at least 50 young people in their counties. After the pilot program, the team came back together to edit and revise the lessons based on teacher input and student evaluation. In 2016, we provided the curriculum to teachers, club leaders, and community members to pilot with their young people. The following surveys analyzed in this study are from this round of pilot tests with individuals from outside West Virginia University Extension teaching the lessons.

The survey was designed with input from the West Virginia University team, West Virginia University Prevention Research Center, and West Virginia Division of Tobacco Prevention. The subjects cannot be identified by the information obtained. Young people were required to take part in the Stop Spit Tobacco lessons in order to take the survey.

The surveys were given to 1,097 students following the completion of the Stop Spit Tobacco curriculum. The survey elicited information about gender, age, and location in the Stop Spit Tobacco lessons. In order to measure the effectiveness of the curriculum with the students, the survey asked them to what extent they agreed or disagreed with a statement based on a four-point Likert scale, with the options ranging from strongly disagree to strongly agree. The statements were:

• I can list some of the ingredients in spit tobacco

• I can explain how trying spit tobacco can lead to addiction

• I can explain how tobacco company ads are misleading

• I can list other things I can buy instead of buying spit tobacco

• I know how to say no to my friends if they offer me spit tobacco

• I know how to get more involved in preventing spit tobacco use at my school or in my community.

## Procedures

1. Surveys were designed with input from the West Virginia University Extension Service, West Virginia Division of Tobacco Prevention, and West Virginia University Prevention Research Center.
2. Upon the approval of the IRB, West Virginia University Extension Service Agents and our community partners asked permission to voluntarily administer the survey to young people who participated in the “Stop Spit Tobacco Curriculum.”
3. Parents received a letter informing them of the project and survey and gave them the option to have their child opt out of the survey. Parents were given at least 2 weeks' notification.
4. Any other Extension Agent (in addition to the PI) who assisted with survey evaluation implementation was trained in CITI-Human Subjects and added to the protocol before survey evaluation administration. Extension agents were also trained in survey evaluation implementation protocols.
5. Young people who completed all six lessons of the West Virginia University Extension “Stop Spit Tobacco Curriculum” (approved by the West Virginia University Prevention Research Center and West Virginia Division of Tobacco Prevention) were asked to complete the survey for the purpose of the program evaluation.
6. Participation in the curriculum did not mandate participation in the survey evaluation. Students had the right to refuse and thus participated voluntarily.
7. Evaluation data from the survey was used to identify the strengths and weaknesses of the curriculum and allow for curriculum revisions. The survey assessed their feelings on spit tobacco use after going through the program.
8. Curriculum instructors were asked to fill out an online survey concerning lesson evaluation. Participation was voluntary.

A total of 1,097 students took part in the 2016 “Stop Spit Tobacco” pilot run across West Virginia. The results of the survey, after the implementation of the curriculum, show that 84.1% of students agree or strongly agree that they can list some of the ingredients in spit tobacco. Ninety-three percentage of student said that they strongly agreed or agreed that they can explain how trying spit tobacco one time can lead to addiction, while 89% of student agreed or strongly agreed that they can explain how tobacco company ads are misleading. Furthermore, 94.7% of students agreed or strongly agreed that they can list other things to buy instead of spit tobacco, and 94.8% students agreed or strongly agreed that they know how to say no to their friends if they are offered spit tobacco. Finally, 85% of the students agreed or strongly agreed that they know how to get more involved in preventing spit tobacco use at their school or in their community. The Table 2 below indicates the self-reported level of knowledge gained after the entire curriculum was complete. This gained knowledge will empower these students to resist spit tobacco in the future and may even provide them with the ability to prevent their friends, family, and community at large from using spit tobacco.

**Table 2:** Evaluation results.

**Please select the appropriate response regarding your level of agreement in accomplishing the following tasks:**					
	**Strongly disagree**	**Disagree**	**Agree**	**Strongly agree**	**Response total**
I can list some of the ingredients in spit tobacco.	4.5% (49)	11.4% (125)	49.1% (538)	35.0% (384)	1,096
I can explain how trying spit tobacco can lead to addiction.	2.3% (25)	4.3% (47)	33.0% (361)	60.4% (661)	1,094
I can explain how tobacco company ads are misleading.	4.8% (53)	6.1% (67)	31.1% (340)	57.9% (633)	1,093
I can list other things I can buy instead of buying spit tobacco.	2.5% (27)	2.8% (31)	16.0% (175)	78.7% (861)	1,094
I know how to say no to my friends if they offer me spit tobacco.	3.4% (37)	2.5% (27)	17.1% (185)	77.0% (835)	1,084
I know how to get more involved in preventing spit tobacco use at my school or in my community.	5.9% (64)	9.1% (99)	40.5% (439)	44.5% (483)	1,085

These are fairly complex topics for students in Grades 3–6 to understand. Following a review of the survey, we know that a large number of students feel they can identify the ingredients of spit tobacco and even explain addiction to it. In regard to identifying misleading spit tobacco advertisements, media literacy is high among young people. Participants know how much spit tobacco costs and can identify better ways in which to spend their money. Many of them report knowing how to say no to their friends, resist peer pressure, and even how to become involved in preventing spit tobacco use at their school and in their community. By educating third-sixth graders on these subjects, we are helping them make healthy decisions in regard to spit tobacco use and to develop essential life skills such as media literacy, leading and promoting a healthy lifestyle, and resisting peer pressure.

## Call to Action for the Cooperative Extension Service

A growing body of research indicates that school-based educational interventions—e.g., interventions that engage elementary and middle school students in interactive educational programs designed around the principles of the “social influence resistance model” to equip young people with key skills for resisting negative social influences—have been shown to be effective in reducing the age of onset and level of tobacco use (CTFK). The final lesson encourages youth to think about how they can get involved with tobacco prevention efforts in their community. Further, youth-led programs that capitalize on these principles of civic engagement have been found to be most effective (20).

The Cooperative Extension System serves as an integral partner in the development and facilitation of interactive educational curricula and programming for elementary and middle schools. As trusted members of their communities, local extension agents are equipped to partner with schools and clubs to provide interactive educational programs in elementary and middle schools. With the ability and expertise to provide effective, short-term, and interactive educational programs for audiences proven to be most receptive to prevention programs, 4-H cooperative extension programs are uniquely poised to help address the need for the development and intervention of research-based, short-term tobacco education programs.

Through the development and delivery of educational programs designed to empower young people to reach their full potential by working and learning in partnership with caring adults, 4-H programs are able to equip and empower young people for healthy, tobacco-free lives. As cooperative extension professionals, we are in a unique situation where we can directly reach young people throughout our schools and communities to prevent the use of spit tobacco. Partnering with local and state partners and using resources such as “Stop Spit Tobacco” will help us to meet our healthy living mission mandate by preventing young people from using spit tobacco. Previous research in West Virginia shows the need for Extension Service leadership in addressing tobacco prevention needs (21).

We encourage our colleagues from across the country to utilize the Stop Spit Tobacco Curriculum in their home counties to help stop the use of spit tobacco. This curriculum will be provided to those seeking it free of charge and can be attained by contacting one of the authors at West Virginia University. As Extension professionals, we are uniquely positioned to provided substance abuse prevention education in our communities, and we should utilize our abilities to do so. Whether it be spit tobacco or any other substance, we are calling on you to take an active approach in substance abuse prevention!

## Data Availability Statement

The original contributions presented in the study are included in the article/supplementary material, further inquiries can be directed to the corresponding author/s.

## Ethics Statement

The studies involving human participants were reviewed and approved by WVU Office of Research Integrity and Compliance. Written informed consent from the participants' legal guardian/next of kin was not required to participate in this study in accordance with the national legislation and the institutional requirements.

## Author Contributions

DR and JR: curriculum development and data analysis. EB: data analysis and manuscript editing. BF-C, BC, NC, ZH, and SL: curriculum development and manuscript development. KD: funder and manuscript development. NR: curriculum development. All authors contributed to the article and approved the submitted version.

## Conflict of Interest

The authors declare that the research was conducted in the absence of any commercial or financial relationships that could be construed as a potential conflict of interest.

## References

[1] National Summit on Smokeless and Spit Tobacco. Overview. 8th National Summit on Smokeless and Spit Tobacco. (2015). Available online at: https://www.sponseasy.com/p/8th-national-summit-on-smokeless-and-spit-tobacco (accessed April 1, 2016).

[2] American Academy of Otolaryngology. Smokeless Tobacco. (2014). Available onine at: http://www.entnet.org/content/smokeless-tobacco (accessed January 26, 2016).

[3] Siddiqi K Shah S Abbas SM Vidyasagaran A Jawad M Dogar O. Global burden of disease due to smokeless tobacco consumption in adults: analysis of data from 113 countries. BMC Med. (2015) 13:1–22. 10.1186/s12916-015-0424-2 26278072 PMC4538761

[4] Centers for Disease Control. Tobacco Brand Preferences. (2020). Available online at: https://www.cdc.gov/tobacco/data_statistics/fact_sheets/tobacco_industry/brand_preference/index.htm (accessed April 13, 2021).

[5] Centers for Disease Control. Economic Trends in Tobacco. (2020). Available online at: https://www.cdc.gov/tobacco/data_statistics/fact_sheets/economics/econ_facts/index.htm (accessed April 13, 2021).

[6] Centers for Disease Control. Smokeless Tobacco: Health Effects. (2014). Available online at: http://www.cdc.gov/tobacco/data_statistics/fact_sheets/smokeless/health_effects/index.htm (accessed February 12, 2016).

[7] American Cancer Society. Health Risks of Smokeless Tobacco. (2015). Available online at: http://www.cancer.org/cancer/cancercauses/tobaccocancer/smokeless-tobacco (accessed February 02, 2016).

[8] U.S. Department of Health Human Services. The Atlanta U.S. Department of Health and Human Services. Centers for Disease Control and Prevention, National Center for Chronic Disease Prevention and Health Promotion, Office on Smoking and Health, Atlanta, GA (2014).

[9] Connolly GN Richter P Aleguas A Jr Pechacek TF Stanfill SB. Unintentional child poisonings through ingestion of conventional and novel tobacco products. Pediatrics. (2010) 25:896–9. 10.1542/peds.2009-2835 20403932

[10] Piano MR Benowitz NL Fitzgerald GA Corbridge S Heath J Hahn E. Impact of smokeless tobacco products on cardiovascular disease: implications for policy, prevention, and treatment. A policy statement from the American Heart Association. Circulation. (2010) 122:1520–44. 10.1161/CIR.0b013e3181f432c3 20837898

[11] Centers for Disease Control. Health Disparities Among Youth. (2020). Available online at: https://www.cdc.gov/healthyyouth/disparities/index.htm (accessed April 13, 2021).

[12] West Virginia Bureau for Public Health. West Virginia State Health Profile 2012. (2013). Available online at: https://dhhr.wv.gov/publichealthquality/statepublichealthassessment/Documents/2012%20State%20Health%20Profile%20Final%20May%202013.pdf (accessed May, 2013).

[13] West Virginia Department of Health and Human Resources Bureau for Public Health Health Statistics Center. Tobacco Use Prevalence Among Youth, West Virginia Youth Tobacco Survey 2013, Charleston, WV (2015).

[14] United States Department of Agriculture. 4-H Healthy Living Logic Model – Prevention of ATOD (Alcohol, Tobacco and Other Drugs). National Institute of Food and Agriculture (2010). Available online at: https://nifa.usda.gov/sites/default/files/asset/document/4-H%20HL-Logic_Model_Alcohol%20Tobacco%20and%20Other%20Drugs_0.pdf (accessed April, 2010).

[15] Campaign for Tobacco-Free Kids. How Schools Can Help Students Stay Tobacco-Free. Tobacco-Free Kids. (2021). Available online at: https://www.tobaccofreekids.org/assets/factsheets/0153.pdf (accessed December 16, 2020).

[16] Centers for Disease Control and Prevention. Guidelines for school health programs to prevent tobacco use and addiction. Morbid Mortal Wkly Rep. (1994) 43:RR-2:12. 8145711

[17] U.S. Department of Health Human Services. Preventing Tobacco Use Among Young People: A Report of the Surgeon General. Atlanta, GA: U.S. Department of Health and Human Services, Centers for Disease Control and Prevention, Office on Smoking and Health (1994).

[18] U.S. Department of Health Human Services. Reducing Tobacco Use a Report of the Surgeon General. Atlanta: U.S. Department of Health and Human Services, Centers for Disease Control and Prevention, National Center for Chronic Disease Prevention and Health Promotion, Office on Smoking and Health (2000).

[19] Centers for Disease Control and Prevention. Tobacco use among middle and high school students—United States, 2011–2016. Morbid Mortal Wkly Rep. (2017) 66:597–603. 10.15585/mmwr.mm6623a1 28617771 PMC5657845

[20] Lantz P Jacobson PD Warner KE Wasserman J Pollack HA Berson J. Investing in youth tobacco control: a review of smoking prevention and control strategies. Tobacco Control. (2000) 47–63. 10.1136/tc.9.1.47 10691758 PMC1748282

[21] Reed D Lester D Danberry K Fink PL Owens S. Perceptions of the role of West Virginia's cooperative extension service in tobacco control coalitions. Front Public Health. (2016) 4. 10.3389/fpubh.2016.00083 27200336 PMC4858527

[22] United States Department of Agriculture. 4-H and Positive Youth Development. National Institute of Food and Agriculture (n.d.). Available online at: https://nifa.usda.gov/program/4-h-positive-youth-development (accessed May 17, 2021).

